# Student Differences in a Social–Emotional Learning Program: Engagement and Individual Factors

**DOI:** 10.1111/josh.70164

**Published:** 2026-05-17

**Authors:** Daijiazi Tang, Lucy R. Zheng, Tyler C. Hein, Jeffrey Albrecht, Natalie Rodriguez‐Quintana, Emily L. Bilek

**Affiliations:** ^1^ Department of Psychiatry University of Michigan Ann Arbor USA; ^2^ Trails Ann Arbor USA; ^3^ Institute for Social Research University of Michigan Ann Arbor USA

**Keywords:** engagement, individual differences, motivation, social–emotional learning

## Abstract

**Background:**

This quality improvement project examined student engagement in a Social–Emotional Learning (SEL) program, analyzing how individual differences such as grade level, gender, and race/ethnicity impacted learning and motivation to use SEL skills.

**Method:**

Post‐program surveys were collected from 981 middle and high school students. Three hierarchical regression models were used to analyze associations between engagement and outcomes: SEL knowledge learned, readiness to use skills, and intention to use skills.

**Results:**

Higher engagement was significantly associated with knowledge learned in SEL skills, readiness and intention to use these skills across most groups. Gender‐diverse students reported lower knowledge or intention than women. Racial/ethnic minority groups reported greater knowledge gains and higher motivation than white students.

**Implications for School Health Policy, Practice, and Equity:**

Continuous quality improvement of SEL curricula that consider students' engagement, developmental stages, and identities may enhance more equitable learning and participation.

**Conclusions:**

Engagement plays an important role in SEL learning and motivation, highlighting the value of developmentally informed approaches that are accessible and relevant to all learners.

## 1 Background

Adolescence, while notable for its opportunities for exploration and learning, is also a vulnerable time for youth, due to rapid physical growth and maturation as well as identity exploration [1, 2, 3, 4, 5]. These critical transitions from childhood to adulthood can negatively impact self‐control and emotional sensitivity [6, 7, 8], leading to behavioral and mental health challenges [5, 9]. Brain development during this period leads to increased urges for risk‐taking long before executive control development catches up [10, 11]. Thus, for some, adolescence can be a time of heightened emotional volatility, relationship stress, mental health concerns, and dangerous behaviors [12, 13, 14, 15].

Social and emotional learning (SEL) programs have been widely applied to help address and prevent these issues. SEL programs aim to help adolescents understand their thoughts and emotions and equip them with effective coping skills, enabling them to handle challenges during this critical period [16, 17, 18, 19]. Developing SEL skills is associated with positive outcomes, such as academic success, healthy relationships, and mental wellness, encouraging some K‐12 education policy changes to expand SEL program implementation [16, 20, 21, 22].

### 1.1 Need for Student‐Centered Evaluation of SEL

Of course, SEL can only support well‐being when youth learn and use the skills. Skill knowledge acquisition (henceforth referred to as knowledge), readiness to use (readiness), and intention to use skills (intention) are important indicators of the success of an SEL program [17, 23, 24, 25].

Research has largely focused on the success of SEL interventions and their implementation from the perspectives of school professionals [17, 26, 27, 28]. This focus is critical, as SEL implementers directly support student social and emotional learning and development [29]. However, student voices are also critical to understand and improve program implementation. Yet, few studies have been grounded in student‐centered investigation of how student engagement in these programs influences SEL knowledge, readiness, or intention to use these skills. Without student‐centered perspectives, researchers and practitioners may miss critical insights about how to support students' SEL development [30, 31].

### 1.2 Learning Engagement and Motivation in SEL Competencies Skills

Engagement in programming enables a deep connection with materials and enhances learning outcomes [32]. Engaged students demonstrate increased retention of information, better problem‐solving skills, and stronger motivation to practice and use skills [33, 34]. In SEL contexts, better engagement supports skill acquisition and practice [35]. Engaged students are likely to connect theoretical knowledge to real‐world scenarios and personal experiences, which leads to more skills practice over time [17, 36].

The influence of SEL engagement on student readiness and intention to use these skills is important. Engaged students directly experience the practical benefits, which reinforces their readiness to apply the skills to various contexts [17, 21]. This can enhance their confidence, leading to proactive use of SEL skills in managing emotions, fostering relationships, and achieving goals.

### 1.3 Individual Differences in Students' Motivation in SEL Competencies Skills

Examining individual differences, such as grade level (e.g., middle school vs. high school), gender, and race/ethnicity, can deepen understanding of students' diverse experiences and needs. Recognizing these differences helps educators align SEL programming with students' developmental and cultural backgrounds, improving the relevance and impact of SEL. These insights also help identify students who may need more customized support or instructional strategies [16, 37].

#### 1.3.1 Motivation in SEL Competency Skills Between Middle and High School

Grade level may be highly relevant to student motivation to SEL programming. Middle school takes place during early adolescence, a time characterized by major emotional and social development [38]. Middle school students often demonstrate higher engagement in SEL activities than high school students [38, 39, 40]. Alternatively, high school students are in middle or later adolescence, where they may be more likely to apply school learnings to their real life [41, 42]. Research indicates that high school students who perceive SEL programs as relevant to their future success are more likely to be motivated and engaged in these activities, whereas middle schoolers engage more when they see immediate social benefits or receive positive reinforcement from others [18, 43, 44]. However, few studies have focused on a comparison between middle and high school students in terms of their SEL engagement and motivation in SEL skills.

#### 1.3.2 Motivation in SEL Competency Skills and Gender Differences

Gender differences have also appeared in SEL research. Girls generally outperform boys in emotion recognition abilities, empathy, and responsible decision‐making [45, 46, 47, 48]. In contrast, boys tend to outperform girls in social engagement and emotional resilience skills [38, 47, 48]. These differences may reflect gender socialization and prior experiences [45, 46, 47, 48, 49, 50]. The experiences of gender‐diverse youth have been overlooked in SEL research. These youth may encounter distinct social–emotional challenges, highlighting the need for SEL programs to be more responsive to the needs of all gender identities [51, 52]. Additionally, few studies have examined gender differences from an integrative perspective in SEL lesson engagement and motivation.

#### 1.3.3 Motivation in SEL Competency Skills and Racial/Ethnic Differences

Understanding SEL learning experiences of racially and ethnically diverse students allows programs to respond to these needs and foster an environment where all students can thrive and develop social–emotional competencies [23, 51, 53]. Some research suggests that Black/African American and Hispanic/Latino students may have less favorable perceptions of SEL, potentially due to disparities in resource availability [54, 55]. However, these differences are not consistently found across racial and ethnic groups, suggesting that the impact of SEL programs may be context‐dependent [30, 53].

Thus, understanding individual differences in students' motivation in SEL skills is crucial for providing empirical evidence and guidance for SEL programs to meet diverse needs.

## 2 The Present Study

To understand youth experiences in an SEL program developed by the TRAILS (Transforming Research into Action to Improve the Lives of Students) organization, this project examined [1] how students' engagement in SEL lessons influenced their learning and motivation in SEL skills, including SEL skills knowledge learned, readiness to use the SEL skills, and intention to use these skills; and [2] how individual differences (grade level, gender, and race/ethnicity) affected these associations. It was hypothesized that student SEL lesson engagement would be positively associated with the three SEL skills outcomes with various levels of individual differences across the links between SEL lesson engagement and SEL skills outcomes (no directional hypotheses posed).

## 3 Methods

### 3.1 Participants

The study included a total of 1093 responses, including 60% middle school students and 37% high school students (3% unknown) from the 6th to 12th grades. Among the participants, 34% identified as female, 36% as male, 5% as non‐binary, 2% as unsure or questioning their gender, and 23% of students did not report their gender. The sample was ethnically diverse, including 1% Alaska Native/American Indians, 1% Asians, 21% Black/African Americans, 3% Hispanic/Latinx, 1% Middle Eastern and North Africans, 14% multiracial, 34% white, and 25% who did not report race/ethnicity.

### 3.2 Instrumentation

#### 3.2.1 SEL Skills Knowledge Learned

Five items were averaged to assess student perceptions of knowledge gained from the SEL curriculum. Each item represented one of the five core CASEL (Collaborative for Academic, Social, and Emotional Learning) skills [56, 57], “Did you learn new things about self‐awareness/self‐management/social awareness/relationship/responsible decision‐making skills in this class?” The items were 4‐point Likert scales ranging from 1 (*I didn't learn anything new about [CASEL skills]*) to 4 (*I learned a lot of new things about [CASEL skills]*). The internal reliability coefficient was satisfactory (*α* = 0.87, *p* < 0.001).

#### 3.2.2 SEL Skills Readiness to Use

Five items were averaged to assess students' perceived readiness to use the SEL skills. Each item represented one of the five core CASEL skills [56, 57], “Do you feel ready to use [CASEL skills] in your life?” The items were 4‐point Likert scales ranging from 1 (*I'm not ready to use these skills*) to 4 (*I'm completely ready to use these skills*). The internal reliability coefficient was satisfactory (*α* = 0.86, *p* < 0.001).

#### 3.2.3 SEL Skills Intention to Use

Five items were averaged to assess students' intention to use the SEL skills. Each item represented one of the five core CASEL skills [56, 57], “Will you use [CASEL skills] in your life?” The items were 5‐point Likert scales ranging from 1 (*I will never use these skills in my life*) to 5 (*I will use these skills all of the time*). The internal reliability coefficient was satisfactory (*α* = 0.86, *p* < 0.001).

#### 3.2.4 SEL Lesson Engagement

Five items were averaged to assess how students engaged in the current SEL curriculum (e.g., “I listened during SEL lessons.” “I stayed on task during SEL activities.”). The items were 5‐point Likert scales ranging from 1 (*Never*) to 5 (*Always*). The internal reliability coefficient was satisfactory (*α* = 0.72, *p* < 0.001).

#### 3.2.5 Demographics

Demographic variables included student self‐reported grade level (6th through 12th), gender, and race/ethnicity. Grade level was categorized to middle school = 0 and high school = 1 to test grade level differences in student SEL learning and motivation.

Gender was categorized to boys (labeled “men” in the survey and henceforth), non‐binary, and unsure/questioning. Girls (labeled “women” in the survey, and henceforth) were selected as the reference group because their SEL development during adolescence is well‐documented and provides a comprehensive baseline for comparison [38]. Women often outperform men in specific SEL skills, such as emotion recognition and empathy, making them a stable and reliable reference point [37, 58, 59].

Race/Ethnicity was categorized to Alaska Native/American Indians, Asian, Black/African American, Hispanic/Latinx, Middle Eastern and North African, and multiracial/ethnicity (white as the reference group). White students are the largest demographic group represented in many educational and SEL studies, providing a baseline for comparison [52]. Additionally, white students were the largest group in this project, enabling reliable analysis with greater statistical power to detect potential individual differences across racial/ethnic groups.

### 3.3 Procedure

TRAILS is a non‐profit organization focused on equipping adults in schools to provide mental health support to students. To foster empathy, self‐awareness, and respect while strengthening academic learning, TRAILS developed a school‐based SEL program offering hands‐on curriculum resources, training, and implementation to support K‐12 classrooms. Grounded in cognitive behavioral therapy (CBT) principles, the TRAILS SEL program is an evidence‐based universal education and awareness curriculum intended to support students' development of SEL competencies. The comprehensive set of 25‐lesson curricula, organized by grade, teaches skills such as challenging unhelpful thinking and fosters the core SEL competencies.

This work was undertaken as a quality improvement (QI) project and did not constitute human subjects research. The project was implemented using the TRAILS SEL program.

Students were recruited through convenience sampling from TRAILS partner middle and high schools in southeast Michigan during Spring 2022. These schools were implementing the TRAILS SEL programming during the 2021–2022 academic year. Classroom teachers administered an anonymous Qualtrics survey to students after they had completed the curriculum.

The anonymous self‐report survey measured student experiences in the SEL program, including demographics, SEL skills, motivation to use CBT skills, and learning interests. This survey was developed with consultation and feedback from SEL experts and schoolteachers.

### 3.4 Data Analysis

Approximately 10% (112) of responses were dropped from 1093 responses for the final analysis due to the missingness of data. RStudio version 2023.12.1.402 (Posit PBC) was used for data analysis with a final sample *N* = 981.

Preliminary analyses included missing data analysis, descriptive statistics, and correlations. Missingness per item ranged from 3% to 17%, showing that the missing data were Missing At Random (MAR). The missingness was partially related to race/ethnicity. Under the assumption of MAR, race/ethnicity was considered appropriate to explain the missingness mechanism, allowing the classification of the current missingness as MAR [60, 61]. This assumption supports the use of multiple imputation to address missing data with unbiased estimations [60, 61, 62]. Multiple imputation with 20 imputed datasets was used for the final analysis to provide unbiased estimates under the MAR assumption.

Three four‐step hierarchical regression models were conducted to examine the effects of SEL lesson engagement, individual differences, and their interactions on students' SEL skills learning and motivation: Knowledge, readiness, and intention. Three sets of regression models examined the outcomes, respectively. SEL lesson engagement was mean‐centered to provide interpretable results by shifting zero to represent the average lesson engagement level [63, 64].

Model 1 introduced SEL lesson engagement. Model 2 introduced grade level (middle school vs. high school) and its interaction with lesson engagement. Model 3 introduced the three categorical gender variables, along with their interactions with SEL lesson engagement. Model 4 included the six categorical race/ethnicity variables and their interaction with SEL lesson engagement. From Models 2 to 4, interaction terms were removed from the final model only if none of the interaction terms between SEL lesson engagement and grade level, gender, or race/ethnicity variables were significantly associated with students' SEL skills learning and motivation.

## 4 Results

### 4.1 Descriptive Statistics

Descriptive statistics are presented in Table 1. Estimates for cell sizes < 10 have been suppressed to protect participant privacy; estimates for cell sizes < 20 have been italicized and labeled as preliminary results due to potential instability of statistical analysis. Significant correlations were found between SEL lesson engagement, learning, and motivation: lesson engagement correlated with knowledge learned (*r* = 0.45, *p* < 0.001), readiness to use (*r* = 0.43, *p* < 0.001), and intention to use (*r* = 0.44, *p* < 0.001).

**TABLE 1:** Descriptive statistics and correlation between SEL lesson engagement and SEL Skills outcomes.

	Mean	SD	Range	Cronbach's *α*	1	2	3	4
SEL lesson engagement	3.38	0.83	1–5	0.72		0.45***	0.43***	0.44***
2 SEL skills knowledge learned	2.73	0.77	1–4	0.87			0.54***	0.52***
3 SEL skills readiness to use	2.88	0.68	1–4	0.82				0.82***
4 SEL skills intention to use	3.62	0.88	1–5	0.82				

	*N*	SEL lesson engagement		SEL skills knowledge learned		SEL skills readiness to use		SEL skills intention to use	
		Mean	SD	Mean	SD	Mean	SD	Mean	SD
Grade level^a^									
Middle school	612	3.40	0.83	2.79	0.73	2.83	0.67	3.55	0.87
High school	354	3.36	0.82	2.61	0.82	2.96	0.69	3.75	0.90
Missing	15	—	—	—	—	—	—	—	—
Gender									
Women	372	3.47	0.82	2.78	0.77	2.95	0.66	3.77	0.82
Men	388	3.39	0.80	2.75	0.73	2.87	0.68	3.61	0.87
Non‐binary	50	3.19	0.78	2.51	0.86	2.66	0.67	3.26	0.84
Unsure/Questioning^d^	16	*3*.*29*	*0*.*79*	*2*.*30*	*0*.*69*	*2*.*62*	*0*.*71*	*3*.*28*	*0*.*88*
Missing	155	—	—	—	—	—	—	—	—
Race/Ethnicity									
AIAN^b^, ^d^	*12*	*3*.*44*	*0*.*85*	*2*.*70*	*0*.*75*	*2*.*80*	*0*.*75*	*3*.*21*	*1*.*08*
Asian^e^	8	*	*	*	*	*	*	*	*
Black/African American	228	3.36	0.78	2.89	0.75	2.94	0.65	3.74	0.87
Hispanics/Latinx	37	3.39	0.75	2.72	0.75	2.77	0.66	3.56	0.89
MENA^c^, ^d^	*13*	*3*.*42*	*0*.*44*	*3*.*03*	*0*.*61*	*2*.*83*	*0*.*65*	*3*.*44*	*0*.*67*
Multiracial	149	3.38	0.84	2.77	0.74	2.59	0.67	3.76	0.74
White	369	3.43	0.84	2.64	0.79	2.86	0.68	3.58	0.90
Missing	165	—	—	—	—	—	—	—	—

*Note*: Note that the final analysis: *N* = 981.

^a^ Grade level included students from the 6th to 12th grade.

^b^ American Indian/Alaska Native.

^c^ Middle Eastern and North African.

^d^ Results for gender‐unsure/questioning, AIAN, and MENA students with 11–19 respondents were italicized and should be considered preliminary.

^e^ Categories with fewer than 10 respondents were suppressed (*) to protect student privacy.

*** *p <* 0.001.

### 4.2 Impacts of SEL Lesson Engagement

Results from Model 1 (Table 2) showed that lesson engagement accounted for a significant proportion of the variance in learning and motivation, *R* ^2^s = 0.19–0.21, *p*s < 0.001. Lesson engagement was positively associated with all three SEL outcome variables (*p*s < 0.001).

**TABLE 2:** SEL lesson engagement predicting SEL skills outcomes.

Independent variables	Knowledge learned				Readiness to use				Intention to use			
	*B*	SE	*R* ^2^	∆ *R* ^2^	*B*	SE	*R* ^2^	∆ *R* ^2^	*B*	SE	*R* ^2^	∆ *R* ^2^
***Model 1***			0.205	0.205			0.188	0.188			0.196	0.196
Intercept	2.73***	0.02			2.87***	0.02			3.62***	0.03		
SEL lesson engagement	0.42***	0.03			0.36***	0.03			0.48***	0.03		
***Model 2***			0.215	0.010***			0.198	0.010***			0.210	0.014***
Intercept	2.78***	0.03			2.82***	0.03			3.54***	0.03		
SEL lesson engagement	0.42***	0.03			0.36***	0.03			0.48***	0.03		
Grade level^a^	−0.16***	0.05			0.14***	0.04			0.22***	0.05		
***Model 3***			0.223	0.008*			0.204	0.006			0.225	0.015**
Intercept	2.81***	0.04			2.85***	0.04			3.62***	0.05		
SEL lesson engagement	0.41***	0.03			0.36***	0.03			0.47***	0.03		
Grade level^a^	−0.16**	0.05			0.14***	0.04			0.20***	0.05		
Gender												
Men	−0.01	0.05			−0.03	0.05			−0.09	0.06		
Non‐binary	−0.17	0.10			−0.18^†^	0.09			−0.39**	0.13		
Unsure/Questioning^d^	*−0*.*40* *	*0*.*18*			*−0*.*22*	*0*.*16*			*−0*.*39* ^*†*^	*0*.*20*		
***Model 4***			0.246	0.022***			0.220	0.017**			0.250	0.025***
Intercept	2.68***	0.05			2.77***	0.05			3.48***	0.06		
SEL lesson engagement	0.42***	0.03			0.36***	0.03			0.48***	0.03		
Grade level^a^	−0.12**	0.05			0.16***	0.04			0.24***	0.05		
Gender												
Men	−0.001	0.05			−0.02	0.05			−0.07	0.06		
Non‐binary	−0.15	0.10			−0.17^†^	0.09			−0.37**	0.13		
Unsure/Questioning^d^	*−0*.*40* *	*0*.*18*			*−0*.*21*	*0*.*17*			*−0*.*33*	*0*.*20*		
Race/Ethnicity												
AIAN^b^	*0*.*07*	*0*.*19*			*0*.*001*	*0*.*19*			*−0*.*27*	*0*.*22*		
Asian^e^	*	*			*	*			*	*		
Black/African American	0.25***	0.06			0.13*	0.05			0.24***	0.07		
Hispanics/Latinx	0.08	0.12			−0.06	0.11			0.02	0.13		
MENA^c^	*0*.*41* *	*0*.*18*			*0*.*09*	*0*.*18*			*0*.*01*	*0*.*22*		
Multiracial	0.15*	0.07			0.13*	0.06			0.24**	0.07		

*Note*: Note that the SEL lesson engagement was mean‐centered.

^a^ Grade level: middle school = 0 (reference group), high school = 1. Gender: Women (reference group), men, non‐binary, and unsure/questioning.

^b^ American Indian/Alaska Native, Asian, Black/African American, Hispanics/Latinx.

^c^ Middle Eastern and North African, Multiracial, and white (reference group).

^d^ Results for gender‐unsure/questioning, AIAN, and MENA students with 11–19 respondents were italicized and should be considered preliminary.

^e^ Categories with fewer than 10 respondents were suppressed (*) to protect student privacy.

*** *p* < 0.001.

** *p* < 0.01.

* *p* < 0.05.

^†^ *p* < 0.10.

**FIGURE 1:**
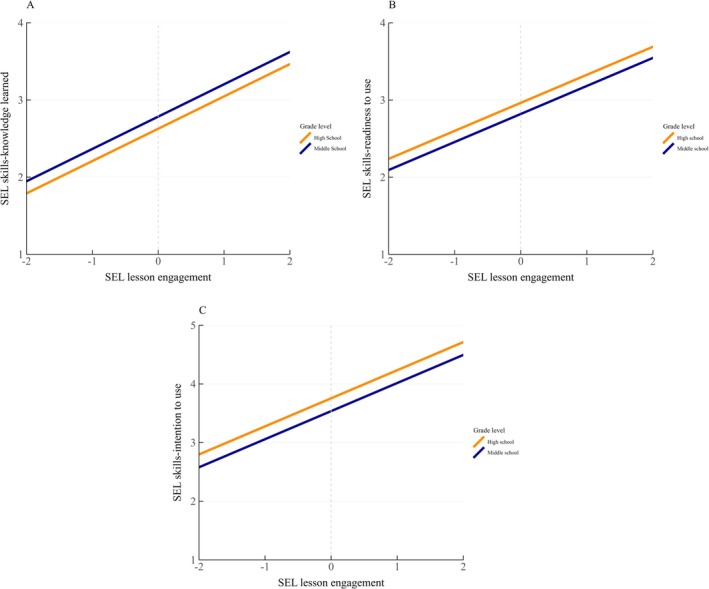
SEL Lesson Engagement Predicting SEL Skills Outcomes by Grade Level. SEL lesson engagement was mean‐centered, showing as the gray vertical gridline. The range from −2 to 2 of the *x*‐axis represents two standard deviations above and below the average of SEL lesson engagement. Full description of the SEL skills‐knowledge learned labels: 1 = *I didn't learn anything new about*, 2 = *I learned a little*, 3 = *I learned some new things about*, and 4 = *I learned a lot of new things about [each of the five CASEL skills]*. Full description of the SEL skills‐readiness to use labels: 1 = *I'm not ready to use*, 2 = *I'm somewhat ready to use*, 3 = *I'm mostly ready to use*, and 4 = *I'm completely ready to use [each of the five CASEL skills]*. Full description of the SEL skills‐intention to use labels: 1 = *I will never use*, 2 = *I will rarely use*, 3 = *I will sometimes use*, 4 = *I will often use*, and 5 = *I will use [each of the five CASEL skills] all of the time*. A significant main effect of grade levels was found on the three outcomes (*p*s < 0.001).

Introducing grade level into Model 2 (Table 2) explained additional variance across all SEL outcome variables, ∆*R* ^2^s = 0.01, *p*s < 0.001. The main effect of grade level was significant across all outcomes while controlling for lesson engagement (Figure 1A–C). High school students were more likely than middle school students to report readiness (*b* = 0.14, *p* < 0.001) and intention (*b* = 0.22, *p* < 0.001). A reverse pattern was found in knowledge, in which middle school students reported they learned more than high school students (*b* = −0.16, *p* < 0.001). The grade level and lesson engagement interaction was not significant and fixed in the following models. Simple slope calculations for each grade level showed that middle and high school students who were more engaged in SEL lessons were likely to learn more and highly motivated to use the skills (slopes = 0.36–0.48, *p*s < 0.001).

**FIGURE 2:**
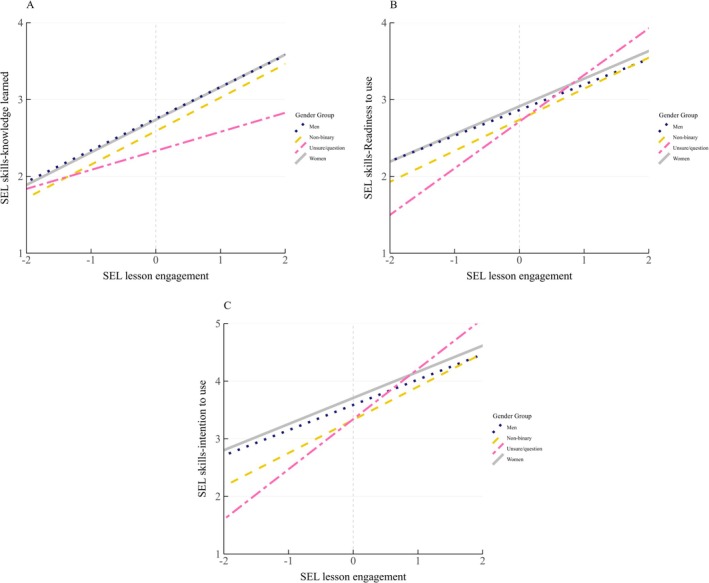
SEL lesson engagement predicting SEL skills outcomes by gender. SEL lesson engagement was mean‐centered, showing as the gray vertical gridline. The range from −2 to 2 of the x‐axis represents two standard deviations above and below the average of SEL lesson engagement. Full description of the SEL skills‐knowledge learned labels: 1 = *I didn't learn anything new about*, 2 = *I learned a little*, 3 = *I learned some new things about*, and 4 = *I learned a lot of new things about [each of the five CASEL skills]*. Full description of the SEL skills‐readiness to use labels: 1 = *I'm not ready to use*, 2 = *I'm somewhat ready to use*, 3 = *I'm mostly ready to use*, and 4 = *I'm completely ready to use [each of the five CASEL skills]*. Full description of the SEL skills‐intention to use labels: 1 = *I will never use*, 2 = *I will rarely use*, 3 = *I will sometimes use*, 4 = *I will often use*, and 5 = *I will use [each of the five CASEL skills] all of the time*. The significant main effects of gender were found on [1] SEL skills‐knowledge learned between women and gender‐unsure/questioning students and [2] SEL skills‐intention to use between women and gender‐non‐binary students (*p*s < 0.05).

Introducing gender into Model 3 (Table 2) explained additional variance for knowledge and intention (∆*R* ^2^s = 0.01–0.02, *p*s < 0.040) but did not explain additional variance for readiness (*p* > 0.13). Gender differences were found in the knowledge and intention variables while controlling for other variables (Figure 2A–C). Unsure/questioning students reported that they learned less than women (*b* = −0.40, *p* = 0.023) and non‐binary students reported lower intention than women (*b* = −0.39, *p* = 0.002). The gender variables and lesson engagement interaction were not significant and fixed for the following models. In terms of simple slopes, lesson engagement was associated with all three outcomes for all gender groups (slopes = 0.25–0.87, *p*s < 0.001).

**FIGURE 3:**
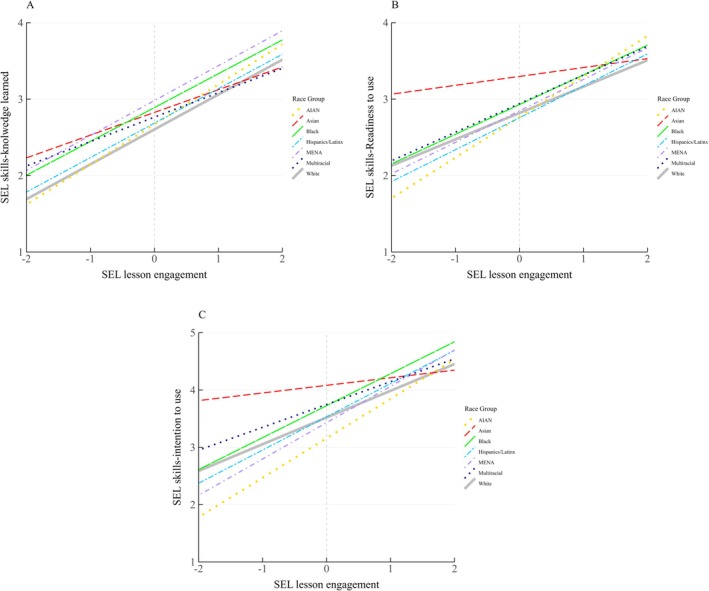
SEL lesson engagement predicting SEL skills outcomes by Race/Ethnicity. SEL lesson engagement was mean‐centered, showing as the gray vertical gridline. Full description of the SEL skills‐knowledge learned labels: 1 = *I didn't learn anything new about*, 2 = *I learned a little*, 3 = *I learned some new things about*, and 4 = *I learned a lot of new things about [each of the five CASEL skills]*. Full description of the SEL skills‐readiness to use labels: 1 = *I'm not ready to use*, 2 = *I'm somewhat ready to use*, 3 = *I'm mostly ready to use*, and 4 = *I'm completely ready to use [each of the five CASEL skills]*. Full description of the SEL skills‐intention to use labels: 1 = *I will never use*, 2 = *I will rarely use*, 3 = *I will sometimes use*, 4 = *I will often use*, and 5 = *I will use [each of the five CASEL skills] all of the time*. The significant main effects of race/ethnicity were found on [1] three SEL skills outcomes between Black/African American and white students, [2] three SEL skills outcomes between multiracial and white students, and [3] SEL skills‐knowledge learned between Middle Eastern and white students (*p*s < 0.05).

Introducing race/ethnicity variables into Model 4 (Table 2) explained additional variance across all three skills outcomes, ∆*R* ^2^s = 0.02–0.03, *p*s < 0.009. Race/ethnicity differences were found across the three outcomes while controlling for other variables (Figure 3A–C). Black/African American, Middle Eastern and North African, and multiracial students were more likely than white students to gain SEL skills knowledge (*b*s = 0.15–0.41, *p*s < 0.028). Black, and Multiracial students reported higher motivation than white students (readiness: *b*s = 0.13–0.59, *p*s < 0.033; intention: *b*s = 0.24–0.71, *p*s < 0.010). Simple slope calculations showed that lesson engagement was significantly associated with all three outcomes across the race/ethnicity groups (slopes = 0.12–0.69, *p*s < 0.03).

## 5 Discussion

### 5.1 Primary Role of SEL Lesson Engagement

As hypothesized, SEL lesson engagement showed significant associations with the three motivation indicators: knowledge learned, readiness to use, and intention to use SEL skills. Regardless of grade level and across most gender and racial groups (omitting subgroups with *n* < 20), higher engagement in SEL lessons indicated stronger motivation to learn and apply SEL skills. Student engagement in programming leads to increased retention of information, better problem‐solving skills, and higher motivation to practice and utilize acquired knowledge [65, 66, 67]. Moreover, SEL studies have found that engaged students are more likely to experience positive outcomes in their academic and personal lives [17, 21, 68].

Engaged students tend to connect theoretical knowledge with real‐world experiences, enabling them to apply SEL skills consistently in various life situations [67, 68]. Our findings align with previous research highlighting the importance of engagement in successful SEL programs. A systematic review suggested that students who actively participated in SEL programs were more likely than less engaged peers to be ready and confident to generalize the skills, ultimately leading to improved academic and social outcomes [17]. Likewise, students who experienced the benefits of SEL skills were more inclined to use these skills proactively, recognizing their value in fostering healthier relationships and achieving personal goals [53, 69].

Thus, promoting learning engagement may be an important strategy to encourage students participating in TRAILS SEL to recall and apply these skills in their daily lives. Engaged students are more likely to internalize SEL skills, which builds confidence and calm when facing social and emotional challenges. This can improve self‐awareness and emotional regulation and strengthen their interpersonal relationships while practicing decision‐making abilities [16, 21, 70].

### 5.2 Individual Differences

As hypothesized, there were individual differences in the three SEL skill outcomes, highlighting the importance of understanding these differences to achieve effective SEL experiences for all students.

#### 5.2.1 Grade Level

Middle school students reported gaining more knowledge of SEL skills than high school students. This reflects developmental stage; students are more likely to build foundational knowledge during the middle school years [38]. In contrast, high school students reported higher readiness and intention to use the SEL skills, reflecting their need to apply these skills in more complex real‐world situations [17]. It is expected that students benefit most when an SEL curriculum is tailored to their developmental needs.

Although the TRAILS SEL program provides a different curriculum for middle and high school students, the findings suggest that the unique needs of middle school students require more attention to focus on knowledge‐based learning and accumulation in the curriculum or applicable practices in the curriculum for high school students. For middle school students, SEL curricula may focus on building foundational skills through structured instruction, basic examples, and reflective activities supporting gradual internalization in familiar settings. For high school students, the curriculum can shift toward application‐ and situation‐based activities to support generalization in real‐world contexts.

Changes can also be made in teacher training to align more closely with students' unique needs: SEL training for middle school staff can center around strategies that facilitate knowledge learning and skills‐building through instruction; training for high school staff and educators can emphasize real‐world applications, equipping educators with techniques to guide students in applying the skills in complex scenarios and broader use.

#### 5.2.2 Gender Differences

In this project, students who identified as women or men reported similar scores in their SEL learning and motivation. This finding is somewhat consistent with existing research, which has shown mixed results regarding gender differences in SEL learning [71, 72]. For instance, studies have found that girls tend to show greater emotion recognition and empathy [45, 46], while boys may show higher social engagement and emotional resilience [37]. This project did not differentiate between knowledge learned in specific SEL skills; however, it is possible that we would have seen similar patterns if the data had been parsed accordingly. In contrast, students who identified as unsure/questioning or non‐binary reported lower levels of knowledge gained or intention to use SEL skills compared to female students, respectively. This novel finding suggests possible gender gaps in SEL programming and highlights a potential area of additional research.

Very few studies have focused on gender‐diverse youth's SEL learning and motivation and their engagement in SEL programs [73, 74]. SEL professionals may have a limited understanding of gender‐diverse student needs and how to adequately support them. Therefore, this finding reflects a general need across SEL programs to focus on gender‐diverse student learning and motivation.

#### 5.2.3 Racial/Ethnic Differences

In our sample, racial/ethnic differences were observed across the three SEL skills motivation indicators. Black/African American, Middle Eastern and North African, and multiracial students reported higher SEL skills motivation compared to white students. These findings are consistent with existing research and contribute to a growing understanding of racial differences in SEL programs [17, 29, 53]. In this sample, white students reported lower learning and motivation compared to some of their peers from other racial/ethnic groups. Several possible explanations may help contextualize this pattern.

Some white students may have been exposed to SEL‐related content through earlier educational experiences or media, leading them to perceive the TRAILS SEL curriculum as less novel. Prior studies suggested that students who encounter familiar or repetitive materials may be less engaged in additional SEL instruction [70]. Alternatively, it is possible that some white students found the content less personally relevant or resonant based on how SEL skills were presented or exemplified. This may potentially limit their connection to the content, especially if the examples, characters, or situations did not reflect their experiences or interests.

Quality of implementation is a critical determinant of SEL program outcomes [68]. We did not examine individual staff variables; however, differences in how the curriculum was implemented could have direct impacts on students' engagement, such as variations in teacher delivery or facilitation styles [68]. For example, some educators may have used more interactive and discussion‐based approaches to encourage reflection and participation, whereas others may have adhered closely to slides or scripts.

These contextual and cultural influences may shape students' experiences in learning SEL curriculum in complex ways. Understanding the roles of content relevance and implementation variability can help interpret differences in students' responses and highlight areas for improvement.

## 6 Implications for School Health Policy and Practice

Our findings highlight the importance of QI projects to support continuous improvement of programming, in this case the TRAILS SEL curriculum. Taking students' prior experiences, identities, and engagement into account may foster improved learning outcomes and meaningful participation for all students. As noted, individual differences may reflect variations in student developmental stages, prior exposure to SEL skills, perceived content relevance, or classroom implementation. Although SEL skills are beneficial for most students, it is important to adopt a culturally responsible approach to better support all students' engagement and motivation in SEL [16, 17, 75].

TRAILS has made improvements and adaptations to the SEL curriculum across the years to provide more individually relevant content. For example, in 2022, TRAILS revised their images and characters to reflect an increased range of cultural elements and depictions of disabilities and assistive devices. Furthermore, throughout training and materials, TRAILS emphasizes the importance of adapting scenarios and lessons to each classroom and encourages staff to adjust the lessons to make the lessons relevant to their students [76]. Recent revisions to the high school curriculum include additional opportunities for student voice and choice, which can enhance engagement and promote connection with the program.

## 7 Limitations

This QI project was not designed to generate findings at a universal level. Accordingly, the interpretations are viewed within the scope of local SEL program refinement. This is not to say that the TRAILS SEL program lacks broader relevance; rather, our findings provide practice‐based insights into students' SEL learning engagement and motivation within real‐world practices. The QI project may serve as a preliminary step for future empirical studies of SEL programs across more representative samples.

The small sample sizes of gender‐unsure/questioning, Asian, American Indian/Alaska Native, and Middle Eastern/North African students in the data limit the generalizability of the findings for these groups. For instance, the gender differences observed among students who identified as unsure/questioning need to be interpreted with caution. The small sample size of students who identified as unsure/questionning (*n* = 16) and American Indian/Alaska Native and Middle Eastern/North African students (*n* < 20) may limit statistical power to detect potential group differences or represent the true experiences of this group. Additionally, we suppressed Asian student statistics to protect their privacy due to the sample size (*n* < 10). The current finding can be considered preliminary evidence, as small subgroup sample sizes limit our ability to fully understand SEL learning experiences within the TRAILS program. Future studies should recruit larger samples and consider alternative sampling strategies and qualitative methods to better capture the experiences and needs of these students.

## 8 Conclusion

This project examined student engagement, motivation, and individual differences within the TRAILS SEL program. The findings highlight the importance of supporting student engagement and refining SEL curricula to better align with school level and the cultural contexts of diverse populations.

## Funding

This work was supported by the Michigan Department of Health and Human Services (MDHHS) and the US Department of Education, Award # U215J180081.

## Ethics Statement

This work was undertaken as a quality improvement (QI) project and did not constitute human subjects research.

## Conflicts of Interest

The authors declare no conflicts of interest.

## Data Availability

The data that support the findings of this study are available from TRAILS. Restrictions apply to the availability of these data, which were used under license for this study. Data are available from the author(s) with the permission of TRAILS.
